# Friends in Our Lives: Perspectives of Young People Who Participated in a Professional Mentoring Program

**DOI:** 10.1007/s43151-025-00189-8

**Published:** 2025-10-27

**Authors:** Kristian Jones, Karryn Satchell, Lakindra Mitchell Dove, Julissa Muñiz, Erik Hulman, J. Mark Eddy, Kevin Haggerty, Deborah Cohen, Martie Skinner, Koren Hanson

**Affiliations:** 1 School of Social Work, University of Washington, Seattle, WA USA; 2 Social Development Research Group, University of Washington, Seattle, WA USA; 3 School of Social Work, Portland State University, Portland, OR USA; 4 School of Education and Information Studies, University of California Los Angeles, Los Angeles, CA USA; 5 Department of Educational Psychology, University of Texas at Austin, Austin, TX USA; 6 Department of Kinesiology and Health Education, The University of Texas at Austin, Austin, USA

**Keywords:** Professional mentoring, Positive youth development, Mentor characteristics, Program feedback, Young people voice

## Abstract

Mentoring programs are popular interventions to support holistic development for young people. One specific model of mentoring that has emerged over the past few decades in the United States is professional mentoring. Research has illustrated that this model of mentoring may be particularly helpful for young people who could benefit from extra support in their lives that a volunteer mentoring program does not have the capacity to provide. Despite the emerging utilization of professional mentoring programs, little is known about the ideal characteristics of a professional mentor and young people’s experiences in these professional mentoring programs. This study interviewed 12 young people who participated in the same professional mentoring program in different regions of the United States. The participants discussed their ideal characteristics in a mentor, highlighted factors that made the professional mentoring program impactful to them, and shared their ideas on how to improve the overall program.

**Supplementary Information:**

The online version contains supplementary material available at https://doi.org/10.1007/s43151-025-00189-8.

Mentoring programs for young people have grown in popularity, both within the United States and abroad, over the past decades. These programs are meant to provide support to young people from various backgrounds and most notably to promote thriving and achieving a variety of positive developmental outcomes (Raposa et al. 2019a; Simpson et al. 2022). Mentoring programs have shown promising results in promoting positive school behavior while also improving prosocial behavior and emotional strength for young people perceived to be at risk for later problematic outcomes (Eddy et al. 2017; Raposa et al. 2019a). Professional mentoring programs for young people, which provide mentors with full-time jobs and provide mentees with long-term supports, have emerged as an option to help support the unique needs of young people facing specific barriers and hurdles in their lives (e.g., poverty, mental health difficulties; Lakind et al. 2014). While the literature illustrates the importance of mentoring and the unique value of professional mentoring, there is a dearth of literature focusing on the perspectives of young people who are in, or have graduated from, professional mentoring programs and the various ways these programs have impacted their lives.

## Professional Mentoring

“Professional” mentors (i.e., mentors employed by a mentoring program) in highly structured programs may be in better positions to partner effectively with marginalized young people and their families (Lakind et al. 2014). Professional mentors serve in long-term, full-time, paid positions with underserved young people perceived to be at risk for problem behaviors and adverse outcomes during adolescence and emerging adulthood. Initially, these young people are engaging with unfamiliar adults, with the goal of developing a supportive relationship over time (Lakind et at.,). Research indicates that most mentors and mentees in these programs are able to develop relationships that continue across time. Entering the mentoring space as a professional can help a mentor gain the credibility and authority they need to promote trust and cooperation among young people, their families, and the other adults in their lives. The impact of professional mentoring has a variety of positive benefits for young people (Eddy et al. 2017) and professional mentors are in the privileged position of being able to serve young people in multiple settings. When organizations keep their caseloads low, they can devote the time needed to help ensure that young people have the comprehensive set of services they need to succeed.

At present, there are few professional mentoring programs in the United States. One of the oldest such programs is Friends of the Children (FOTC). FOTC requires mentors to have relevant education and experience working with young people before making a match and then requires ongoing specialized training in order for mentors to provide the best possible services to young people and families. Within the professional relationship role, the literature suggests that establishing a strong, close interpersonal connection between the mentor and mentee is essential to furthering the young person’s social-emotional, cognitive, and identity development (Lakind et al. 2014; Eddy et al. 2017). FOTC is an especially unique program, as it utilizes a long-term (12 plus years) approach designed to create ongoing, meaningful experiences that empower young people to build life skills and make healthy choices while also exploring and promoting a young person’s diverse talents and interests (How It Works 2024). In the context of FOTC, the term professional can be conceptualized as mentors that commit to at least 3 years of service as a mentor to provide relationship continuity for the young person in the program as a salaried employee focused full time on mentoring about 8 young people on their caseload (Keller et al. n.d.).

Mentors, known as “Friends” are carefully selected, trained, and supervised to support young people across their development until they graduate from high school. Before children enter the program, they often have experienced systemic obstacles and complex trauma. Each child enrolled in the program is guaranteed a mentor for 12 years or through high school graduation. FOTC is hypothesized to work by bolstering social capital and encouraging young people to delay family formation, complete their secondary education, and avoid involvement in antisocial behaviors and thus contact with the juvenile justice system. These specific outcomes are rooted in theories like Positive Youth Development theory (Lerner et al. 2017) and Emerging Adulthood (Arnett 2007) that programs in the United States often emphasize as necessary for young people to become contributing members of society. The program emphasizes the development of core values and skills aimed at helping young people achieve these outcomes. Currently, FOTC reaches thousands of children attending over 650 schools located in 37 different communities within the United States (How It Works 2024).

## Positive Youth Development Framework

The Positive Youth Development (PYD) theory proposes that with guidance and support from caring adults, young people have the potential to thrive and make positive contributions to not only their own development but also to their families, schools, communities, and society as a whole (Lerner et al. 2017). According to this theory, developmental assets that promote optimal development for young people consist of key experiences and people necessary to enhance a young person’s developmental experiences (Lerner et al. 2014). Thus, nurturing and supportive adults are among the most critical assets for promoting positive youth development (Lerner et al. 2014) and professional mentors are uniquely situated to help young people reach their full potential to contribute as much as they can to society. This framework underscores the many ways mentoring programs—and specifically professional mentors can—promote positive youth development and act as barriers against risk factors linked to negative psychological, behavioral, and problematic behaviors (DuBois et al. 2011; Raposa et al. 2019a).

Despite the acceptance of this framework by both researchers and practitioners, there is a lack of literature that identifies the ideal characteristics of mentors needed to facilitate positive youth development from the perspective of the young person receiving mentorship. Further, insights on ways to improve youth development programs such as mentoring programs from the perspectives of young people who have had time to reflect on their time in their respective programs have been understudied in youth development research but are critical in ensuring programs are both centered around the young person and promote social justice (Bloomer et al. 2023; Osai et al. 2024).

## Current Study

The study’s purpose is to illuminate the experiences of young people who participated in a professional mentoring program. The central aims of the study are to investigate what characteristics allowed the participants to build rapport with their mentor and examine how young people perceived the benefits they gained because of participating in a longitudinal mentoring program. A secondary aim is to explore potential recommendations the young people may have to improve the program after having some time to reflect on their experiences after completing the program. This study is part of a larger quantitative and qualitative study that explored the perceptions of young people who participated in FOTC as mentees, and one aspect of an ongoing longitudinal randomized clinical trial of FOTC (Keller et al. n.d.; Eddy et al. 2017).

## Methods

### Procedure

Participants were originally selected from a randomized clinical trial (RCT) study of FOTC that began in 2007 (see Eddy et al. 2017) and, based on when the young person entered first grade, were expected to graduate from high school in the spring of 2020. Purposive sampling included individuals who indicated on their last round of surveys of the RCT that they were open to being contacted for follow-up qualitative interviews. Twenty-nine of the 41 consented to future follow-up interviews, with seven participants not meeting eligibility criteria. Of the 29, only participants (*n* = 22) from the intervention condition were eligible to participate in the qualitative interview. Attempts to equally distribute gender and geographic location were made when prioritizing who to contact first from the list of 22, and ultimately, 12 (55%) young people responded and were interviewed. We had three young people from each of the four regions of the country where the FOTC RCT took place. The qualitative component of this study was considered by the relevant university’s Institutional Review Board (IRB) and was determined to be exempt. Informed consent procedures were followed for all the young people who participated in the interviews as well.

At the time of the study, participants were between the ages of 20 and 21. Most young people identified as Black (58%), with the remainder of the participants identifying as Latine (17%) or White (17%), with a few identifying as other (8%). Sixty-seven percent of the participants identified as women. The participants were from four FOTC programs, one in Washington State, one in Oregon, one in New York, and one in Massachusetts. Fifty percent of the participants in this study were from the Oregon chapter and were part of the original, and largest, FOTC program. The average amount of time spent in the program for this subsample was 8.67 years, with 33% receiving a “full dose” of 12 or more years in the program. The majority of our sample (58%, *n* = 7) reported that they had received at least 10 years of the program. Interviews ranged between 60 and 90 min in length. Semi-structured interview guides covered a range of topics including the selection process to be included in the program, the various developmental stages of the program from elementary to high school, post-secondary preparation, mentorship experiences, and their perceptions about the program.

### Data Analyses

The researchers utilized a rapid qualitative analysis approach (RQA; Keniston et al. 2023; Nevedal et al. 2021). RQA typically involves immediate summarization using templates and/or matrices, identifying key themes and drawing conclusions quickly (Hamilton and Finley 2019). This allowed the research team to focus on the verbatim transcripts and efficiently analyze the participants’ quotes, with the intent of gathering the information quickly in order to disseminate it back to the participants themselves and to FOTC staff as well as inform real-time intervention development and quality control. All interviews were audio recorded using the video-conferencing platform, Zoom, and were transcribed using the professional version of Otter.ai. Once reviewed and edited for spelling and auditory processing errors, the transcripts were organized into a matrix template spreadsheet that contained the guiding questions and broader topics from the interview guide (see Appendix). Three researchers from the research team then coded each transcript by reading through each one and placing quotes from participants into the matrix template and analyzing the content of each matrix (Keniston et al. 2023; Nevedal et al. 2021). Any conflicts in thematic categorization were addressed via majority vote among the research team. Once all transcripts had been coded by interview guide topics in their matrices, the researchers met to distill recurring themes and subthemes from the matrix templates and solidify the results from the analysis. RQA was considered appropriate for this study as findings had to be disseminated back to community partners and the participants themselves quickly in order to receive feedback and inform future FOTC program delivery (Keniston et al. 2023).

## Results

Coding of participant responses yielded 3 themes: Desired Characteristics in a Mentor, Gaining Exposure and Connections through the Program, and Feedback to Improve FOTC. The themes are described below.

### Desired Characteristics in a Mentor

According to the young people interviewed, specific interpersonal skills were beneficial when forming a successful mentoring relationship. Many of the participants listed interpersonal skills that promoted rapport and ultimately led to a close connection with their mentor, such as charisma, sense of humor, empathy, friendliness, patience, consistency, and being reliable. Many of the young people stressed the importance of being engaging and catching the young person’s attention, particularly in the beginning of the relationship to help the young person feel comfortable. A mentee (#1) explained “…because if you’re boring, your kids are not gonna like you. You got to have a sense of humor.” In addition to those characteristics, participants also identified the importance of their mentors empowering and supporting them throughout the relationship no matter what their goals were during the relationship. One young person (#2) explained how encouragement was a common trait exhibited throughout the relationship from the various mentors he had:

But some similarities to all of them…they always wanted to help in anything that I wanted to do if I wanted to find a new career choice. Or if I had interest in choice, or if I had interest in learning new things, they always were there to help me with that.

Ultimately all the young people reported that mentors must be understanding and patient with their mentees, even if the mentees are making mistakes. One former mentee (#9) detailed:

You have to be understanding, even if you haven’t gone through the same situation….try your best not to get frustrated with your kid. Because the more you get frustrated with them, the less likely they are to open up about the things that you want them to open up about.

Participants also identified the importance of mentors’ role modeling certain skills to them such as communication, emotional regulation, and problem solving. For example, the same mentee (#9) reflected, “Yeah, it got me to be around other people help with my social skills help with my anxiety, it helps me see things like differently, like, Oh, hey, you know, this is how you communicate with other people, not the way that I saw it in my house.”

Additionally, many participants expressed how having a mentor with a similar racial and/or ethnic background was also helpful in developing rapport and trust in the mentoring relationship. One former mentee (#8) spoke about her experiences with sharing the same language and background with her mentor; she went on to explain, “…[my mentor] had a Spanish background, so we could just relate on a lot of different things. And it just felt good having somebody who I can relate to and talk to.” Another young adult (#11) expressed similar sentiments in regard to connecting to one of their mentors more than another, “I think I could connect to people who looked like me a little bit more than how I could connect with [another mentor].” Feeling as though you were understood by your mentor appeared to be the most frequent comment made by mentees when they had well-established rapport with their mentor. Promoting mentee’s individualized development through relatable characteristics, modeled skills, and shared similar life experiences was supported by mentee’s comments.

In regard to the interpersonal skills identified by the participants that were essential for building strong relationships with their mentors, those identified by participants echo previous research findings in the youth mentoring literature (Deutsch and Spencer 2009; Goldner and Ben-Eliyahu 2021; Spencer 2006). Specifically, participants mentioned a variety of ideas that were identified in Spencer’s (2006) influential study on the relational processes necessary for building strong bonds between the mentor and the mentee. In particular, participants identified the importance of empathy, authenticity, collaboration, and companionship. Additionally, participants’ emphasis on mentors making the relationship engaging and catching the young person’s attention aligns with qualitative research that has highlighted that both male and female mentees expect to engage in fun and interesting activities with their mentor throughout the relationship (Spencer et al. 2018). Lastly, other youth mentoring research has underscored the importance of patience and flexibility among mentors providing youth mentoring (Deutsch and Spencer 2009; Thomson and Zand 2010). The findings illustrate that certain interpersonal skills and general characteristics appear to be universal among different types of mentoring programs and interventions.

Participants also identified benefits of having mentors that shared similar cultural, ethnic, and/or racial backgrounds as them. Specifically, participants expressed that having someone who shared a cultural identity made it easier to connect deeper on certain topics. Other youth mentoring research has also highlighted benefits of having mentors who share the cultural, ethnic, or racial background as their mentee. Specifically, Raposa and colleagues (2019b) found ethnic and racial similarity between young people and their mentors predicted matches that lasted longer. Overall, these findings further reinforce the importance of interpersonal skills that cultivate connection that can develop into a high level of relationship quality between young people and their mentors (De Wit et al. 2020).

### Gaining Exposure and Connections Through the Program

Young adults identified opportunities and activities that they engaged in while in FOTC that they believe they would not have had access to without the program. Some of these activities were leisure activities that were meant to be fun for the participants. A young person (#11) described all the activities they were able to engage in while in FOTC,

...we did a lot of camping trips and stuff. They also did snowboarding that I wasn’t really interested in. But everybody that went said that they liked snowboarding. We’ve gone to Storm [WNBA] games…Seahawks [NFL] games, Mariner [MLB] games. [we] have that done a lot.

Another young person (#4) described being able to go to an overnight camp for the first time in his life and the lasting impact it had on him, “…it’s a whole new environment, like you’re just stuck in a city, but then the guy who takes you to the camp, I think it’s like, somewhere upstate, I believe, and just doing a whole bunch of new things.” The same participant went on to explain, “I feel like I’m able to share experiences that I’ve had [at camp], that actually stick, like swimming there. Anytime I swim, I think about how I wouldn’t be able to swim if it wasn’t for [my mentor].”

The young people also described having the space and opportunity to explore new and different career paths. For example, one former mentee (#2) recalled, “…And one time I had an interest in advertising, and I got a chance to go to downtown Portland and get an interview at a bank and ask them questions about [advertising].” Another young person (#8) discussed how helpful the program was for career development and thinking about life after high school.

[the program] opened up so many opportunities for me, helping me write my resume and creating a LinkedIn profile, helping me find opportunities. I remember they took us on a couple college tours, we went to one in New York and it was such a fun experience…

Participants explained that these types of opportunities were common in FOTC due to mentors tailoring activities to their specific interests. As one young person (#11) shared, “…And if I had interest in things, then my mentor would try and find little things to do that was towards that interest.”

While most participants discussed the benefits of having support with applying for college and scholarships, going on college tours, and being able to discuss the college experience with their mentor or other FOTC staff members, some participants shared that they did not attend college following high school and noted that while participating in the program there was emphasis on college preparation. One young person (#1) reported that “Friends of the children helped me get into my dream school when I graduated. But like I said, I didn’t go.” Another participant (#11) shared her interest in the medical field, but no desire to be in school for a long time, so she decided to enroll in a program to be an ultrasound technician. Another participant (#5) reflected on how it would have been beneficial to have a vision along the way, possibly through shared stories of what other participants experienced. “… maybe if I saw more kids that have been through the program, to give me feedback, like, this is what I was going through, and this is what helped me and what I’m doing now… you know, give me a vision along the way of what could be, you know?”. These participants emphasized other ways to gain exposure to potential career paths that did not exclusively focus on a 4-year university and ways in which preparation for the transition to emerging adulthood would have been helpful.

Most participants also mentioned that having access to financial assistance and equipment, provided by the program, allowed them to expand their knowledge and engagement with different skills. One former mentee (#1) recounted,

...When I was editing a video for one of my wrestling teammates, he was sending it to his colleges…I was like I don’t have an iPhone. I don’t have an Apple computer. Is it okay if I use the Macbooks at Friends? And [the program said] yeah, come in whenever you want.

Another young person (#9) was inspired by their experience in the program, so much so that they now wanted to be a caseworker themselves, stating, “… [the program] made me want to be a caseworker, growing up being in that program working with [my mentor] seeing what she did, it made me want to give back, do that for a kid.” Potentially as a result of access to different activities and opportunities, a few of the participants discussed how participating in these new endeavors encouraged them to be more open-minded in other areas of their life. One participant (#3) credited FOTC for his willingness to engage in new experiences as an adult; he stated,

“... the program actually helped me to go outside and try new things. Everybody says it now…you go out and try new things, which now that I’m thinking back on it, it was because of the program that I'm like that. [the program] was a huge factor of why I'm like that right now.”

The same participant continued,

I think that was a huge takeaway for me to be able to see the problems that I have inside myself, and to give me the courage and like I said [earlier], the perseverance to be able to change things about myself that I wouldn’t have known to do otherwise.

The positive impacts of the activities and opportunities provided by FOTC were echoed by most of the participants. It’s evident in listening to the young people’s recollections that having the freedom to explore and try new interests and activities with the support of their mentor was influential to their development. One of the participants (#5) put it best, “I learned it’s ok to be myself.” Lasting impacts on interpersonal skills, development, and preferences were commonplace among the young people interviewed.

According to participants, one way the positive youth development framework was evident in the FOTC mentorship experience was how mentors exposed participants to new and expansive experiences to foster the positive youth development of the young people in the program. Specifically, FOTC mentors cultivated positive youth development through providing participants access to activities and career development opportunities that they may not have had the opportunity to engage in otherwise. The importance of activities in the mentoring relationship and the definition of such experiences according to personal experiences and context (e.g., gender, age, and setting) has been identified previously in the mentoring literature (Lakind et al. 2015; Nakkula and Harris 2010). The participants’ insights also illustrate the cultivation of social capital (i.e., the importance of relationships and connections to different types of groups and the potential resources that come with those relationships and connections; Barker 2012) and the importance of exposing young people to various activities and experiences in and outside of their neighborhood. These findings corroborate other mentoring literature that has highlighted that mentoring relationships can be a powerful tool in cultivating social capital for young people (Gowdy and Spencer 2021).

In contrast, the benefits of instrumental mentoring (i.e., mentoring focused on cultivating and/or teaching specific skills) have been mixed. Some empirical evidence highlights that programs with structured activities and targeted mentoring approaches were associated with higher effect sizes when compared to mentoring programs without such characteristics (Christensen et al. 2020; DuBois et al. 2002). Other studies indicate that the benefits of instrumental mentoring are not as clear as those of mentoring relationships that are focused on cultivating the emotional connection between the young person and the mentor (e.g., Goldner and Ben-Eliyahu 2021). Findings from this study add to the youth mentoring literature by highlighting the potential long-term benefit of using mentoring program activities to both expose program participants to new activities and to help them with educational and career goals and expand their social capital (Gowdy and Spencer 2021).

### Feedback to Improve FOTC

Despite the general positive experiences reported by participants, several shared recommendations for how FOTC could improve. There were three consistent topics related to the recommendations: (1) wage equity for mentors, (2) improving the transition process when young people were assigned new mentors, and (3) transparency around the program selection process. Further, a couple of young adults discussed having to deal with their assigned mentor’s biases and prejudices in the relationship. Young adult participants talked at length about wage equity for their mentors. One young person (#10) declared, “[Mentors] should get paid a little bit more than they do. I know it’s hard work and it’s like having four or five more kids.” Another young person (#1) explained how a conversation with a former mentor shed light on the inequities:

Based off my experience, my conversations now [on] LinkedIn, and I asked [former mentors], how did they like it? Because they’re no longer working [at FOTC]. They’re like, “honestly, the equity for the staff was not good.” So, my feedback for [FOTC] is to do better work, not just for your youth, but for your employees.

Other participants expressed similar concerns for their mentors’ financial well-being and felt that higher wages might prevent mentors from eventually quitting. When mentors quit, mentees are often left feeling abandoned and/or confused, which speaks to the other theme we consistently heard regarding the sometimes tumultuous and abrupt transition from one mentor to another.

Several participants shared that they wish the transitions from one mentor to another were handled in a more transparent, understanding, and communicative manner. A majority of the participants in the study had at least three or four different mentors while enrolled in the program over a 12-year period. Many of them discussed how hard it was to transition to another mentor after having developed such a strong bond with their former mentor. For example, one former mentee (#6) expressed how hard it was initially to transition from the first mentor to the next mentor, although they eventually developed a solid relationship:

…it’s definitely nothing like that first connection that you have with the first person that came to your school and that bond that [was] created. So, it was definitely hard opening up to a new mentor, because I’ve already gotten very close with [my first mentor]... it wasn’t the same, but it was definitely to a point where I felt comfortable with [my second mentor].

Similarly, another young person (#8) shared the following about their transition:

When she told me she was leaving. I cried. Because she was supposed to be with me for longer. She was supposed to be with me up until the end of my senior year … things were just hard for her. And I was like, I totally understand, take care of yourself... but I was so sad. It was so hard for me to say goodbye.

Additionally, participants felt that the transition was difficult in part because of the lack of communication and time to prepare for the change in mentors. One former mentee (#12) described:

… I had just reached the summer going into my 6th grade year … I had to transition to my third mentor because of the age limit...And it was a very short notice. I didn’t know that [I had to switch mentors]. So, when I found out, I was like, “oh, okay, I’ve met this person maybe once or twice that I got transitioned with. And I wasn’t ready to [switch mentors].

One young person (#2) shared how news of a mentor leaving the program would get discussed throughout the organization and resulted in young people in the program hearing about mentor transitions even when it was not their assigned mentor:

But whenever somebody would leave Friends of the Children, it would become a huge topic of discussion. And [the remaining program staff] would make me feel a certain way, “Like, oh, well, that person was so nice. Like, why are they leaving? Is there something wrong with friends?” So, I feel like they could probably keep that kind of stuff a little bit more confidential.

In addition to reflecting on the emotional difficulties and the confusion that often accompanied switching mentors, participants also shared tangible suggestions on ways to improve upon the transitioning process between one mentor to another. One of the participants (#6) suggested a built-in transition period. Specifically, she described how her current mentor brought her upcoming mentor along on events to ease the transition:

Before [My first mentor] had switched over she brought [my second mentor] along with us to our events that way I can kind of get used to her. She kind of brought me in on it slowly, so [my second mentor] kind of already knew how, like, the type of person I was.

Moreover, when discussing difficulties with the mentor transitioning process and agency support for their mentors, almost unilaterally, each young adult mentioned a lack of understanding about why they were specifically selected to be in the FOTC program in the first place. For context, children are selected when they are 4–6 years old, and the conversation about why they are being invited to participate in FOTC is had with the caregiver, not with the child due to the child’s young age. One young person (#5) expressed confusion when first meeting their mentor, “…it was interesting, because, when I met my mentor, I didn’t know what was going on.” Another former mentee (#2) echoed similar sentiments, declaring, “I have no idea (why they joined the program). And I’ve never asked that question to anyone.” One young person (#4) shared that they assumed they were in the program due to their quiet demeanor but were never told why exactly they were selected for the program. He explained, “I can’t remember (the first interactions with the mentor). It was a while ago … thinking about it … [my mentor] is probably speaking to me, because I’m shy, I’m quiet.” Another young person (#11) described what it was like to deal with why others thought they were in the program as a child:

It felt like they were saying like I needed [my mentor] to get through school, or like I needed help with something like education, or like I had anger problems or something, which I didn’t, but I don’t know, it just made me feel like they were like saying this mentor is like a tutor slash social worker or something that was helping me fix my life when it wasn’t necessarily like that. It was more so like a friend that’s there for you no matter what it is. And so, balancing the differences with what I knew and what people were assuming it was a little challenging in the beginning.

In addition to not understanding why they were enrolled in FOTC, a few participants shared specific scenarios where their mentors would bring their own unchecked biases and prejudices with them into the mentoring relationship. Specifically, two young people shared stories about their mentors passing judgment on their weight during the mentoring relationship. One young person (#10) detailed, “She would make me cry. But I guess now that I’m older, I just felt like it was unreasonable, you’re making a third grader, fourth grader go on a diet like, [I’m] still a kid.” Another former mentee (#7) had similar experiences with a mentor in the program stating,

I go from one mentor to another mentor, [mentor’s name], she’d always take us out for ice cream, cake or sweets or something like that. And then I got somebody later, she was very, very, aware of my weight and tried to make me very self-aware of my weight, like very self-conscious about it. And so, I did become very self-conscious at one point about my weight when I was little.

The same participant reflected that mentors have a great deal of power in the mentoring relationship and how they choose to work with the young person is important, “… [mentors] got to understand you … be willing to work with you and grow and not point out your insecurity and kind of use them against you or kind of bully you or manipulate you into thinking that you are the problem.” Overall, the constructive feedback provided highlights the importance of soliciting feedback from young people participating in the program and conducting ongoing evaluations throughout the entirety of the program.

Despite the many positive experiences expressed by participants, many of them identified several areas of improvement for FOTC. Specifically, former participants identified the importance of wage equity for their mentors, improving the transition process when young people were assigned new mentors, transparency around the program selection process and dealing with their assigned mentor’s biases and prejudices. Participants identifying wage equity as a pivotal issue for their mentors aligns with previous youth mentoring studies that emphasize that lower-income volunteer mentors tend to have shorter mentoring relationships compared to their peers, highlighting that not having adequate financial resources may make this line of work difficult to carry out (Grossman and Rhodes 2002). It is important to acknowledge that wage equity is a critical issue in the public sector in the United States, especially in non-profit human services jobs (Cunningham et al. 2017; Wage Equity Study Team 2023) and is also a significant issue when considering job satisfaction for younger workers across the globe (Filandri et al. 2023). To try to combat this, FOTC uses a market-based, composite salary indicator that is inclusive of starting teacher and social worker salaries in a chapter’s market. Ongoing cost of living and merit increases, as well as full benefits, are also provided by FOTC. Finally, addressing the transition of mentoring relationships is of the utmost importance, as research illustrates that youth mentoring relationships that are terminated prematurely or rematched can cause harm to young people (Grossman et al. 2012).

Young people in this study also identified the lack of clarification as to why they were selected into the program in the first place as an issue. This seemed to increase the young person’s sense of being “othered” based on some unknown fault or issue. To the authors’ knowledge, this is the first study to highlight former participants of a mentoring program expressing a desire to have more clarification as to why they were selected to participate in the program. This finding illustrates the need for youth development programs to be clear and transparent as to why young people are selected for a program because lack of clarity may cause confusion on the young person’s end and potentially be a significant barrier in promoting positive youth development in the young person’s life. One way that programs can be transparent as to why young people are selected for a youth development program would be thinking of developmentally appropriate ways to talk with young people as to why they are selected to be in the program and how the program intends to collaborate with them and their families, and to have this discussion along various points in development.

Lastly, a couple of the young people who participated in the study expressed feeling judged by their mentors, specifically in regard to their diet and weight. Importantly, promoting healthy choices, as it is often referred to, is a part of FOTC’s core goals when working with young people in the program. It is possible that while striving to promote healthy choices among young people in the program, mentors could have come across as insensitive or judgmental. It is also possible that these specific negative interactions between young people and their mentors are a byproduct of unaddressed implicit biases, specifically class bias. Spencer and colleagues (2022) have found that social class bias can negatively impact the mentor–mentee relationship. Further, Albright and colleagues (2017) highlight that mentoring programs can often be used to provide low-income young people with middle-class mentors with the expectation that low-income young people will automatically benefit from mentorship from someone from a higher social class. The participants’ insights on their experiences of feeling judged illustrate the importance of both mentoring programs and individual mentors being more self-aware of their prejudices and biases, so they do not unintentionally harm the young people they intend to serve, particularly as they try to implement the goals of the program into action and truly promote positive youth development without imposing middle-class values among marginalized young people. The findings also underscore the importance of ongoing training and self-reflective practice for mentors serving young people. Professional mentors may be in a unique position to receive the preparation and ongoing programmatic support needed to be constantly growing as mentors and as human beings to best serve ever-evolving young people.

### Practice Implications for Youth Development Organizations

The results of this study provide several pertinent implications for both youth mentoring programs and broader youth development programs. First, findings illustrate the importance of mentoring programs, along with other institutions serving young people, to focus on wage equity, being transparent about why the young person is in the program, and the potential for mentor transitions. Specifically, the findings highlight the need for programs to be mindful of how it transitions a young person to a new mentor and supporting the mentor with resources (e.g., cultural sensitivity training, financial support) to give them the best opportunity to serve young people (Jones et al. 2023b; Sánchez et al. 2021; Spencer et al. 2020). Youth mentoring programs and other institutions serving youth should also consider how to promote and train mentors to be more culturally sensitive and aware of their own biases. Another strategy might be to include important people in the young person’s social networks in the mentoring intervention if they are unable to match young people to mentors with shared racial, ethnic, or cultural identities (Hagler et al. 2023; Jones et al. 2023a; Sánchez et al. 2021). In doing so, it may help mitigate some of the potential harm and cultural mismatch some young people described in their qualitative interviews.

Furthermore, the insights from the young people underscore the significance of being intentional with the type of activities (e.g., college tours, camping trips) programs expose participants to, as these activities can leave a lasting impression. Their experiences also highlight the potential drawbacks to focusing mainly on 4-year universities as the next and main step for the transition to emerging adulthood. Whether this is a result of program objectives, aspirations of the mentors and their hopes for mentees, or a combination of both, it is important that mentees are involved in developing a viable transition plan that is expansive and not limited to higher education as the sole option, rather exploring multiple pathways that could support the transition into emerging adulthood based on the young person’s interests, passions, skills, and capacities. Finally, findings illustrate the importance of mentors being mindful of their own biases and prejudices (e.g., body weight shaming; Carter et al. 2023 and social class bias) and being mindful of the young person’s wellbeing when attempting to reach positive outcomes to make sure they are not passing their own judgment onto the young people they are supposed to be uplifting.

## Limitations

Although this study adds a great deal of insight into youth mentoring and broader youth development literature, it is not without limitations. First, the qualitative nature of this study limits generalization. Second, the authors only interviewed young people who participated in four FOTC chapters. FOTC is a nationwide program with locations across the United States, and young people in different types of youth mentoring programs may have different experiences and insights to share that do not align with the themes presented in this study. Furthermore, recall bias could have played a role in perceptions of the program. Since FOTC is a longitudinal mentoring program, it is possible that the young people who recall memories from their childhood could be biased. Lastly, some of the participants did not complete the FOTC program all the way through high school graduation for various reasons (i.e., moving to a different state), which is the intended amount of time to spend in the program. Their perceptions of the program may be different from young people who were in the program throughout the intended amount of time.

## Conclusion

Young people reported a variety of different elements of the program that they enjoyed, and a few components that could be improved upon. Many of the participants reflected on the helpful characteristics, interpersonal skills, and shared experiences of their mentors, as well as the opportunities being in FOTC allowed them to take advantage of during various points in their lives. Often, participants recalled the novelty of many of the experiences they had and exposure to activities they would not have otherwise had access to and/or encouragement to engage in. Feedback was offered on critical aspects of the program, such as wage equity for mentors, the transition process to a new mentor, more transparency as to why they were enrolled in the program to begin with, and more awareness from mentors on sensitive topics. Overall, participant insights add to the youth development literature and illustrate the potential impact, positive and negative, that a longitudinal program can have on the life of a young person.

## Supplementary Information

Below is the link to the electronic supplementary material.

Supplementary Material 1 (DOCX 16.7 KB)

## Data Availability

Original deidentified transcripts are available from the first author.
