# Supplementary material for: Friends in Our Lives: Perspectives of Young People Who Participated in a Professional Mentoring Program
Source: J Appl Youth Stud. 2025 Oct 27;9(3):471–89. doi: 10.1007/s43151-025-00189-8 (PMC13042457; doi:10.1007/s43151-025-00189-8)
Supplement: Supplementary file 1 [file 43151_2025_189_MOESM1_ESM.docx]

Appendix

Sample Interview Guide

**Warm-up: Background Information**

1. When did you first join the Friends of the Children?
   a. How did you first learn about Friends of the Children?
2. When you think about Friends of the Children, what comes to mind? If someone asked you to describe the Friends of the Children program, what would you say?
3. Besides having a mentor/Friend, what is Friends of the Children all about?
4. What, if anything, do you recall about the early memories of being a part of the program?
   a. Why do you think you were invited to participate in this program?
5. How do you remember how the program and/or mentorship process explained to you?
6. What was the communication between the Friends of the Children and you/your family like while you were in the program?
   a. Are there areas of potential improvement?
7. What barriers, if any, got in the way of you being able to participate in Friends of the Children?

**The Mentorship Experience**

*The next set of questions will specifically dig into your experience with mentorship during the 12 years you participated in Friends of the Children*

1. How many different mentors/Friends did you work with?
   a. If more than one, can you tell me a little about each one? How long did you work with each Friend?
   b. How often did you and your mentor/Friend meet? How did this change as you grew up?
   c. Where would you typically meet?
2. What would you say is the role of the mentee?

*For these next questions, you can speak to different mentors/Friends or choose one or two that you would like to focus on.*

3. Can you tell me about the first time you met your very first mentor/Friend? What was that initial meeting like?
4. If you had multiple mentors/Friends, can you tell me a bit about each one?
   a. How did your relationship with each one vary? And why?
5. Did you share any similarities with your mentor/Friend? (Probes: Racial identity, sex other factors) If so, how did that impact, if at all, the nature of your relationship with them?
6. What are different things that your mentor/Friend did to connect and build a relationship with you?
   a. What worked?
   b. What didn’t work?
7. What are some things that you’ve done with your mentors/Friends over the years?
   a. What are your most memorable experiences with a mentor/Friend?
8. What qualities do you think are needed to be a good mentor/Friend?
   a. Can you give me an example of a time you felt supported by your mentor/Friend?
   b. Are there other ways that you wish your mentor/Friend would have supported you?
9. What did it mean to you to have a mentor/Friend growing up?
10. Which of your mentors/Friends did you connect with the most? And why?

**Reflections during Young Adulthood**

1. Now that the program is over, looking back, in what ways do you think your life is different because you were in Friends of the Children?
2. What feedback do you have about the program based on your experiences?
   a. What about the program is really good – that really needs to keep happening?
   b. What was not a part of mentorship that you wish you could have experienced through the program?
   c. How could you have been better supported?
   d. Is there anything else that is really important for the program to do that it isn’t doing now?
3. What are some things you’ve learned from being in Friends of the Children that you’ll take with you into this next stage in life?
4. Do you think you and your mentor(s)/Friend(s) will stay in touch? Why or why not?
5. Do you have any closing thoughts or reflections that you would like to share? What else do you think is very important for people to know about what is like to participate in Friends of the Children?
